# First Detection and Genomic Characterization of Linezolid-Resistant *Enterococcus faecalis* Clinical Isolates in Bulgaria

**DOI:** 10.3390/microorganisms13010195

**Published:** 2025-01-17

**Authors:** Tanya V. Strateva, Preslava Hristova, Temenuga J. Stoeva, Hristina Hitkova, Slavil Peykov

**Affiliations:** 1 Department of Medical Microbiology “Corr. Mem. Prof. Ivan Mitov, MD, DMSc”, Faculty of Medicine, Medical University of Sofia, 2 Zdrave Str., 1431 Sofia, Bulgaria; 2 Department of Microbiology and Virology, Faculty of Pharmacy, Medical University of Pleven, 1 St. Kliment Ohridski Str., 5800 Pleven, Bulgaria; preslavahristova@outlook.com (P.H.); hitkova@abv.bg (H.H.); 3 Department of Microbiology and Virology, Faculty of Medicine, Medical University of Varna, 55 Marin Drinov Str., 9002 Varna, Bulgaria; temenuga.stoeva@abv.bg; 4 Department of Genetics, Faculty of Biology, University of Sofia ‘St. Kliment Ohridski’, 8 Dragan Tzankov Blvd., 1164 Sofia, Bulgaria; 5 BioInfoTech Laboratory, Sofia Tech Park, 111 Tsarigradsko Shose Blvd., 1784 Sofia, Bulgaria

**Keywords:** *Enterococcus faecalis*, linezolid resistance, whole-genome sequencing, G2576T mutation, *optrA*, pAR0780 plasmid, hospital-adapted ST6 lineage, ST1102

## Abstract

Linezolid is an oxazolidinone antibiotic and is considered a last-resort treatment option for serious infections caused by problematic Gram-positive pathogens, including vancomycin-resistant enterococci. The present study aimed to explore the linezolid resistance mechanisms and genomic characteristics of two vancomycin-susceptible *Enterococcus faecalis* isolates from Bulgaria. The strains designated Efs2503-bg (inpatient from Pleven) and Efs966-bg (outpatient from Varna) were recovered from wounds in 2018 and 2023, respectively. Antimicrobial susceptibility testing, whole-genome sequencing, multilocus sequence typing, and phylogenomic analysis based on 332 linezolid-resistant *E. faecalis* genomes were performed. Efs2503-bg was high-level resistant to linezolid (MIC > 256 mg/L) and displayed the G2576T mutation affecting three of the four 23S rDNA loci. Efs966-bg (MIC = 8 mg/L) carried a plasmid-located *optrA* determinant surrounded by *fexA* and *ermA*. No mutations in the genes encoding for ribosomal proteins L3, L4, and L22 were detected. The isolates belonged to the sequence types ST6 (Efs2503-bg) and ST1102 (Efs966-bg). Phylogenomic analysis revealed that Efs2503-bg and Efs966-bg are genetically distinct, with a difference of 12,051 single-nucleotide polymorphisms. To our knowledge, this is the first report of linezolid-resistant enterococci in Bulgaria. Although the global incidence of linezolid-resistant enterococci is still low, their emergence is alarming and poses a growing clinical threat to public health.

## 1. Introduction

*Enterococcus* species (mostly *Enterococcus faecalis* and *Enterococcus faecium*) are ubiquitous Gram-positive bacteria that usually inhabit the intestinal tract of healthy humans and animals, but are also found in other anatomical sites, including the vagina and oral cavity, as well as in soil, water, plants, and fermented food products [1,2,3,4]. In addition to their role as commensals, enterococci are considered a major cause of opportunistic healthcare-associated and community-acquired infections worldwide [5,6]. They may be responsible for various infections, including urinary tract infections, bacteremia and sepsis, endocarditis, burn and surgical site wound infections, abdomen and biliary tract infections, device-associated infections, and others [7].

The severe infections caused by *Enterococcus* spp. are hard to treat due to the adaptability of the organisms to the hospital environment, their intrinsic resistance to commonly used antimicrobial drugs, including cephalosporins, aminoglycosides (low-level resistance), clindamycin, and sulfonamides, and in the case of *E. faecium*, low-dose penicillin and ampicillin, as well as their great capacity to acquire mobile genetic elements carrying antimicrobial resistance (AMR) genes, and a variety of virulence factors [5,8]. Since the first reports of vancomycin-resistant enterococci (VRE) in the 1980s, epidemiological studies have demonstrated the rapid expansion, considerable morbidity, high mortality, and the economic burden of VRE-associated infections globally [9,10,11]. Therefore, the importance of vancomycin-resistant *E. faecium* has been recognized by the World Health Organization, which classified it among the high-priority pathogens for the research and development of new antibiotics [12].

The synthetic oxazolidinone linezolid (LNZ) is one of the last-resort treatment options for serious infections caused by multidrug-resistant (MDR) Gram-positive pathogens, including VRE, such as nosocomial and community-acquired pneumonia, complicated skin and soft tissue infections, and even bloodstream infections. LNZ binds in the V domain of the 23S rRNA component of the 50S ribosomal subunit, thus inhibiting polypeptide synthesis and elongation [13,14,15]. Although previous studies have indicated that LNZ resistance is uncommon, resistance rates in clinical *Enterococcus* spp. isolates have been increasing over the last decade [16,17,18,19].

Several specific mechanisms have been reported to be associated with linezolid-resistant enterococci (LRE). The major mechanism in clinical LRE isolates is attributable to point mutations in 23S rDNA alleles. The most common nucleotide exchanges are G2576T (*Escherichia coli* numbering) and G2505A [20]. Alterations in the L3, L4, and L22 ribosomal proteins are related to decreased LNZ susceptibility [21,22]. Additionally, transferable LNZ resistance determinants, such as *cfr*, *cfr(B)*, *cfr(D)*, *optrA*, and *poxtA*, have been found in *Enterococcus* spp. The plasmid-borne multidrug resistance gene *cfr*, which encodes an rRNA methyltransferase, was first described in an animal *Staphylococcus sciuri* isolate and was thereafter reported in various Gram-positive and Gram-negative bacteria of diverse origin [23,24]. It confers resistance to phenicols, lincosamides, oxazolidinones, pleuromutilins, and streptogramin A antibiotics (PhLOPSA phenotype) [25]. In addition, *cfr*-like genes such as *cfr(B)* and *cfr(D)*, have been identified in clinical *Enterococcus* isolates [26,27,28]. However, the contribution of *cfr*-like genes to reduce the LNZ susceptibility in enterococci is still under debate [29]. The *optrA* gene encodes an ATP-binding cassette F (ABC-F) protein that is responsible for transferable resistance to oxazolidinones and phenicols [30,31]. Originally, *optrA* was identified in Chinese *E. faecalis* and *E. faecium* isolates of human and animal origin but was subsequently detected in LRE worldwide [32,33]. The *poxtA* gene encodes a protein that is 32% identical to OptrA and possesses structural features typical of the ABC-F protein superfamily that cause antibiotic resistance through ribosomal protection [34]. It confers a reduced susceptibility to oxazolidinones, phenicols, and tetracyclines, and has been found in clinical LRE in Europe [35,36,37].

The present study aimed to explore the LNZ resistance mechanisms and genomic characteristics of two vancomycin-susceptible *Enterococcus faecalis* isolates obtained from Bulgarian patients with wound infections, compared with 332 genomes of LNZ-resistant *E. faecalis* strains from around the world.

## 2. Materials and Methods

### 2.1. Bacterial Strains and Clinical Case Presentation

Two LNZ-resistant *E. faecalis* isolates obtained from infected wounds of Bulgarian patients were included in the present study.

The first *E. faecalis* Efs2503-bg isolate (designated Efs2503-bg) was recovered at the beginning of July 2018 from a surgical wound of a 19-year-old male inpatient who underwent surgical treatment at the Clinic of Orthopedics and Traumatology of the University Hospital “Dr. Georgi Stranski” (950 beds) in Pleven. In late May 2018, the patient was admitted to the Emergency Department with polytrauma due to a motor vehicle accident, where he underwent wound debridement and direct skeletal extension of the left lower extremity. After the stabilization of his general condition, external fixators were placed on the left femur and left tibia and a debilitating direct skeletal extension was performed. Due to developing traumatic pulmonitis, the patient was transferred to the Intensive Care Unit. Three weeks later, in good general condition, he underwent surgical interventions including blood reposition and the metal osteosynthesis of two of the fractures. The postoperative period was complicated by a surgical site infection (SSI) which required the placement of a drainage. The surgical wound sample was positive for *E. faecalis* (Efs2503-bg) and *Pseudomonas aeruginosa*. During the long hospital stay before SSI was diagnosed, the patient was treated with a combination of antibiotics, including LNZ.

The second *E. faecalis* Efs966-bg isolate (designated Efs966-bg) was obtained in September 2023 from a wound secretion of a 53-year-old male outpatient from Varna with type 2 diabetes mellitus and a diabetic ulcer. He was also diagnosed with hypertensive disease, diabetic polyneuropathy, and chronic arterial vascular insufficiency of the lower extremities. The patient had not been admitted to a hospital in the last year. There was no information available on recent antibiotic treatment. The wound sample was also positive for *E. coli* and *Candida albicans*.

*E. faecalis* ATCC 29212 was used as a control strain for species identification, antimicrobial susceptibility testing, and resistome analysis.

### 2.2. Species Identification

Species identification was accomplished using the BD Phoenix M50 automated system (BD, Franklin Lakes, NJ, USA). The identification of Efs2503-bg and Efs966-bg was confirmed by analyzing the assembled draft genome sequence using the Microbial Genomes Atlas (MiGA) Web server [38]. The workflow was carried out with default settings.

### 2.3. Antimicrobial Susceptibility Testing

Antimicrobial susceptibility testing (AST) of the two *E. faecalis* isolates studied was performed by the disk diffusion assay and the minimum inhibitory concentration (MIC) gradient method (MIC Test Strip; Liofilchem, Roseto degli Abruzzi, Italy), according to the European Committee on Antimicrobial Susceptibility Testing (EUCAST) version 14.0, 2024 guidelines, with the following antimicrobial agents: LNZ, ampicillin, imipenem, gentamicin (detection of high-level aminoglycoside resistance (HLAR)), tigecycline, eravacycline, levofloxacin, vancomycin, and teicoplanin [39]. The antimicrobial disks were manufactured by Oxoid (Basingstoke, UK). As broth microdilution (BMD) is considered the reference method for AST of rapidly growing aerobic bacteria [40], LNZ MIC values were additionally assessed using the MIKROLATEST MIC plates (Linezolid 16–0.12 mg/L; Erba Lachema, Brno, Czech Republic) according to the manufacturer’s instructions. The EUCAST clinical breakpoints for *Enterococcus* spp. were applied to interpret the results obtained. In particular, resistance to LNZ is defined as > 4 mg/L, whereas an MIC ≤ 4 mg/L is considered as susceptibility.

### 2.4. Definition of MDR E. faecalis Isolates

According to previously described criteria [41], MDR *E. faecalis* isolates are non-susceptible to at least one agent in three or more categories, including aminoglycosides except streptomycin (high-level gentamicin resistance), streptomycin (high-level resistance), carbapenems (imipenem), fluoroquinolones (ciprofloxacin, levofloxacin), glycopeptides (vancomycin, teicoplanin), glycylcyclines (tigecycline), penicillins (ampicillin), and tetracyclines (doxycycline, minocycline).

### 2.5. DNA Isolation

Total DNA from the investigated isolates was obtained by the ZymoBIOMICS DNA Miniprep Kit (Zymo Research Corp., Irvine, CA, USA), according to the manufacturer’s instructions. Extractions were performed from 3 mL of overnight cultures inoculated with a single colony and grown in Brain Heart Infusion medium (HIMEDIA^®^, Kelton, PA, USA).

### 2.6. Whole-Genome Sequencing (WGS)

The two LNZ-resistant *E. faecalis* isolates were subjected to WGS in order to analyze their resistomes and virulomes. The extracted genomic DNA was randomly fragmented, size-selected, and ligated to adapters, followed by PCR amplification. Subsequently, the generated libraries were sequenced on an Illumina NovaSeq 6000 platform (Novogene, Cambridge, UK), generating 2 × 150 bp paired-end reads.

### 2.7. Draft Genome Assembly

The applied workflow comprised multiple steps as follows: quality control (FastQC v0.11.9, https://www.bioinformatics.babraham.ac.uk/projects/fastqc/, accessed on 27 December 2024), raw read preprocessing (Trimmomatic v0.38) [42], genome assembly (SPAdes v3.12.0) [43], and draft genome metrics evaluation (Quast v5.2.0) [44]. All software tools were accessed through the Galaxy online platform [45] and were executed with default parameters unless otherwise specified.

### 2.8. Resistome Analysis

Both assembled draft genome sequences were screened for AMR genes using ABRicate (v1.0.1) with the following settings applied: Comprehensive Antibiotic Resistance Database (CARD) [46], minimum DNA identity (70%) and minimum DNA coverage (60%). A second search with the same settings was applied against the PlasmidFinder Database to identify all plasmid replicons in the assembled contigs.

In addition, the ribosomal proteins L3, L4, and L22 were manually inspected for amino acid exchanges related to LNZ resistance. Correspondingly, the coding sequences of *gyrA* and *parC* were examined for sequence variations within their quinolone resistance-determining regions (QRDR). Both analyses were performed using blastn comparisons with the corresponding sequences in the *E. faecalis* ATCC 29212 strain (CP008816.1)

Finally, the pre-processed sequencing reads originating from both isolates were mapped against the full-length 23S rDNA of *E. faecalis* ATCC 29212 using Bowtie2 [47]. The resulting BAM files were subsequently examined for mutations through visual inspection with the Integrative Genomics Viewer (IGV) [48].

The read-mapping against the pAR0780 plasmid (CP063981.1) was performed by applying the Bowtie2 tool and the result was visualized by the BLAST Ring Image Generator (BRIG) program [49].

### 2.9. Virulome Analysis

The assembled genomes of Efs2503-bg and Efs966-bg were screened for virulence determinants using the VFanalyzer tool (https://www.mgc.ac.cn/cgi-bin/VFs/v5/main.cgi, accessed on 21 December 2024) available at the Virulence Factor Database (VFDB) [50], employing default parameters.

### 2.10. Multilocus Sequence Typing (MLST)

The Galaxy MLST tool (Version 2.19.0, https://usegalaxy.eu/, accessed on 27 December 2024) was used to perform MLST analysis. The utilized *E. faecalis* MLST scheme incorporates internal fragments of the following seven housekeeping genes: *gdh* (glucose-6-phosphate dehydrogenase), *gyd* (glyceraldehyde-3-phosphate dehydrogenase), *pstS* (phosphate ATP binding cassette transporter), *gki* (putative glucokinase), *aroE* (shikimate 5-dehydrogenase), *xpt* (shikimate 5-dehydrogenase), and *yiqL* (acetyl-coenzyme A acetyltransferase) [51].

### 2.11. Phylogenomic Analysis

Existing LNZ-resistant *E. faecalis* isolates with genomes deposited in the National Center for Biotechnology Information (NCBI) Nucleotide database (*n* = 382 as of 1 October 2024) were identified using the search terms “*Enterococcus faecalis*”, “*linezolid resistant*”, and “*linezolid resistance*”. Their corresponding sequences underwent quality evaluation with Quast v5.2.0 and CheckM v1.2.3 [44,52]. All draft genomes with a total number of contigs (>1000 bp) greater than 200, less than 98.5% completeness, or more than 1% contamination were excluded from the subsequent analysis. The remaining nonduplicate genomes (*n* = 332) were screened for AMR genes and subjected to MLST analysis via the above-mentioned tools. Upon completion, all sequences were annotated using Prokka v1.14.6 [53]. Then, the pangenome pipeline Roary v3.13.0 [54] was employed to generate a core gene alignment with a minimum blastp identity of 95% and a core definition threshold of 99%. The SNP-sites tool was used to extract all single nucleotide polymorphisms (SNPs) from the resulting multi-FASTA alignment file, and a SNP distance matrix was constructed for all isolates. Following matrix sorting, the 50 strains most closely related to each of our isolates were identified. Finally, phylogenetic trees were constructed based on their core gene alignments using the RAxML program (v8.2.12) with the neighbor-joining clustering method and 1000 bootstrap replicates [55]. The Interactive Tree Of Life portal was employed to create a graphical representation of the phylogenetic tree [56].

## 3. Results

### 3.1. Antimicrobial Susceptibility

The AST profiles of the two *E. faecalis* isolates studied are presented in Table 1. The nosocomial Efs2503-bg isolate was classified as MDR. It showed high-level LNZ resistance (above 16 mg/L), HLAR, resistance to levofloxacin (>32 mg/L), as well as non-susceptibility to imipenem (1 mg/L). The Efs966-bg isolate (outpatient) was categorized as resistant to LNZ (8 mg/L), intermediate to imipenem (0.75 mg/L), and susceptible to all other antibiotics tested. As shown in the table, both isolates were susceptible to glycopeptides, tigecycline, and the new fluorocycline eravacycline.

### 3.2. Draft Genome Assemblies: Evaluation and Comparison

The two assembled draft genomes showed sizes of 3.18 Mb and 2.97 Mb, respectively, with a GC content of approximately 37.2% (Table 2). These values are comparable with the accessible data from sequenced *E. faecalis* genomes. Also, Efs2503-bg was ascribed to the sequence type (ST) 6 (included into the high-risk clonal complex (CC) 2), whereas Efs966-bg belonged to the ST1102.

### 3.3. WGS-Based Resistome Analysis

**Figure 1:**
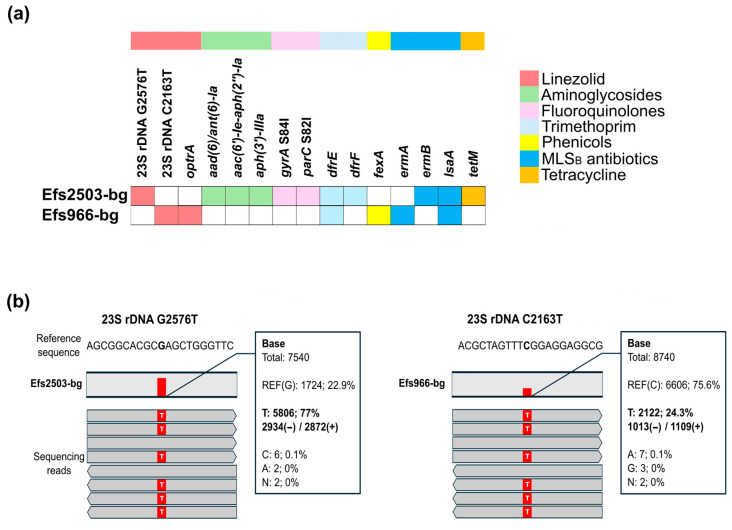
Resistome analysis of two linezolid-resistant *E. faecalis* isolates obtained from Bulgarian patients. (**a**) Matrix with all identified AMR-related mutations and determinants, identified in the two sequenced genomes. MLS_B_ antibiotics, macolide–lincosmaide–streptogramin B antibiotics. (**b**) Mapped reads statistics for both identified variants in the 23S rDNA. The values were obtained by the IGV browser.

All identified AMR determinants in the two sequenced genomes are illustrated in Figure 1a. Resistome analysis revealed that Efs2503-bg carries a G2576T mutation in three of the four 23S rDNA loci, as determined by sequencing read coverage that contributes to the observed LNZ resistance (Figure 1b). A blastn search revealed that this variant was found in only two complete (CP004081.1 and CP019512.1) and three draft genomes in the NCBI database (GCF_002105365.1, GCF_002105335.1, and GCF_016400905.1). In contrast, the Efs966-bg genome exhibited a C2163T mutation affecting only one 23S rDNA locus (Figure 1b). No missense variants were detected in the genes encoding the ribosomal proteins L3, L4, and L22 in either isolate.

As shown in Figure 1a, the nosocomial Efs2503-bg isolate possessed several genes encoding aminoglycoside-modifying enzymes (AMEs), including the *aac(6′)-Ie-aph(2″)-Ia* (HLAR except streptomycin) and *aad6/ant(6)-Ia* (high-level streptomycin resistance) genes [57,58]. Comparison of the QRDRs of both isolates with the corresponding regions of *E. faecalis* ATCC 29212 revealed that the Efs2503-bg isolate owned two amino acid substitutions (p.S84I in *gyrA* and p.S82I in *parC*) with a role in fluoroquinolone resistance.

**Figure 2:**
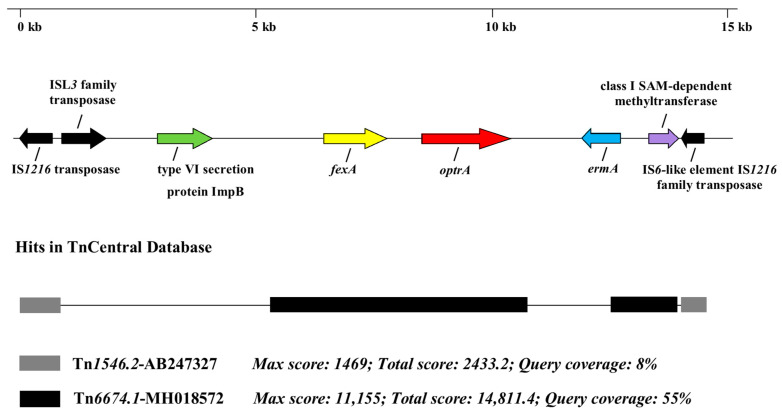
Schematic representation of the *optrA* environment in the *E. faecalis* Efs966-bg isolate. The figure also shows the presence of antimicrobial resistance-associated genes and mobile genetic elements, as well as the best matches for the region in the TnCentral database.

The Efs966-bg isolate obtained from an outpatient was positive for the *optrA* gene. Manual analysis of the genetic environment identified several genes associated with AMR and mobile genetic elements located both upstream and downstream (Figure 2). A comparative BLASTn analysis of the entire sequence in the TnCentral database [59] revealed various hits, with Tn*6674.1*-MH018572 and Tn*1546.2*-AB247327 showing the highest max and total scores.

**Figure 3:**
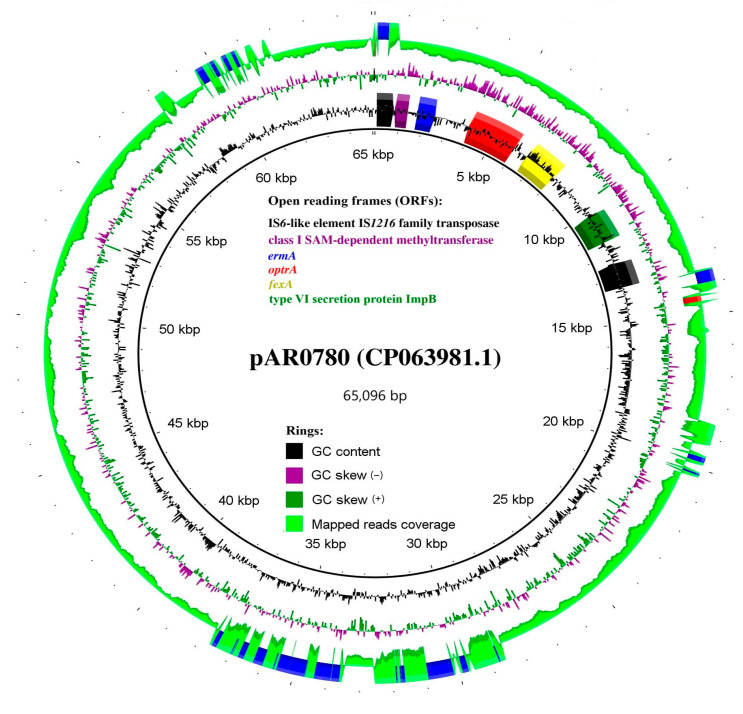
*E. faecalis* pAR0780 plasmid, showing read-mapping from the sequencing reads, CDS, and mobile genetic element-related genes. Alignments were performed using BLAST+. The image was generated using BRIG. CDS: coding sequences.

The *optrA* gene was located on a contig measuring 64,749 bp, with sequencing coverage 2.3 times higher than that of contigs containing genes with known chromosomal locations. This coverage disparity suggested a plausible plasmid localization. This hypothesis was confirmed by identifying the full-length rep9b_4_repA2(pTEF2) replicon on the same contig. Blast search with the entire sequence generated a nearly perfect hit (99% query coverage and 100% identity) against the *E. faecalis* plasmid pAR0780 (CP063981.1, length: 65,096 bp). Mapping the sequencing reads against its sequence showed that Efs966-bg also possessed the remaining plasmid elements, as illustrated in Figure 3. In total, 99.78% of the bases in the sequence of pAR0780 were covered with a 4436x average depth. Only a short region between positions 14,366 and 14,549 was absent.

### 3.4. WGS-Based Virulome Analysis

**Figure 4:**
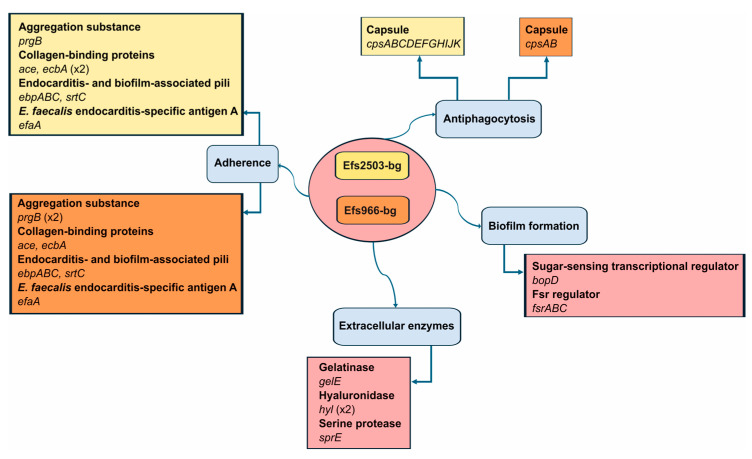
Virulome analysis of two linezolid-resistant *E. faecalis* isolates obtained from Bulgarian patients. All identified virulence-associated genes are presented and grouped according to the classes defined in the Virulence Factor Database (VFDB).

Both isolates carried a repertoire of virulence-related genes encoding proteins involved in adherence, antiphagocytosis, biofilm formation, and extracellular enzyme production. Particularly, this includes several genes encoding adhesins (*prgB*, *ace*, and *efaA*) and invasins (*gelE* and *hyl*). The complete list is provided in Figure 4.

The major difference between the studied LNZ-resistant isolates was the presence of nine *cps* genes in the genome of the MDR nosocomial Efs2503-bg isolate, related to capsule formation. Furthermore, one of the two *prgB* genes identified in Efs966-bg was located on the detected pAR0780 plasmid.

### 3.5. Phylogenomic Analysis of Linezolid-Resistant E. faecalis Strains

To examine the epidemiology of the investigated isolates, a set of 332 nonduplicate genomes of LNZ-resistant *E. faecalis* was collected as previously described. They were subjected to MLST and resistome analyses (Supplementary Table S1). A total of 70 different STs were detected, with ST480 (19% of the isolates) and ST16 (13.6% of the isolates) being the most prevalent. Notably, neither ST6 nor ST1102 were detected among the 332 genomes associated with the MLST affiliation of our *E. faecalis* isolates. The *optrA* gene was identified in 293 genomes, making it the most prevalent determinant of LNZ resistance in this group by a great margin. Eight of the isolates harbored *poxtA*, two were positive for *cfrB*, and *cfrA* was spotted only once. It is worth mentioning that only one of the 332 isolates was found to carry a determinant associated with vancomycin resistance.

**Figure 5:**
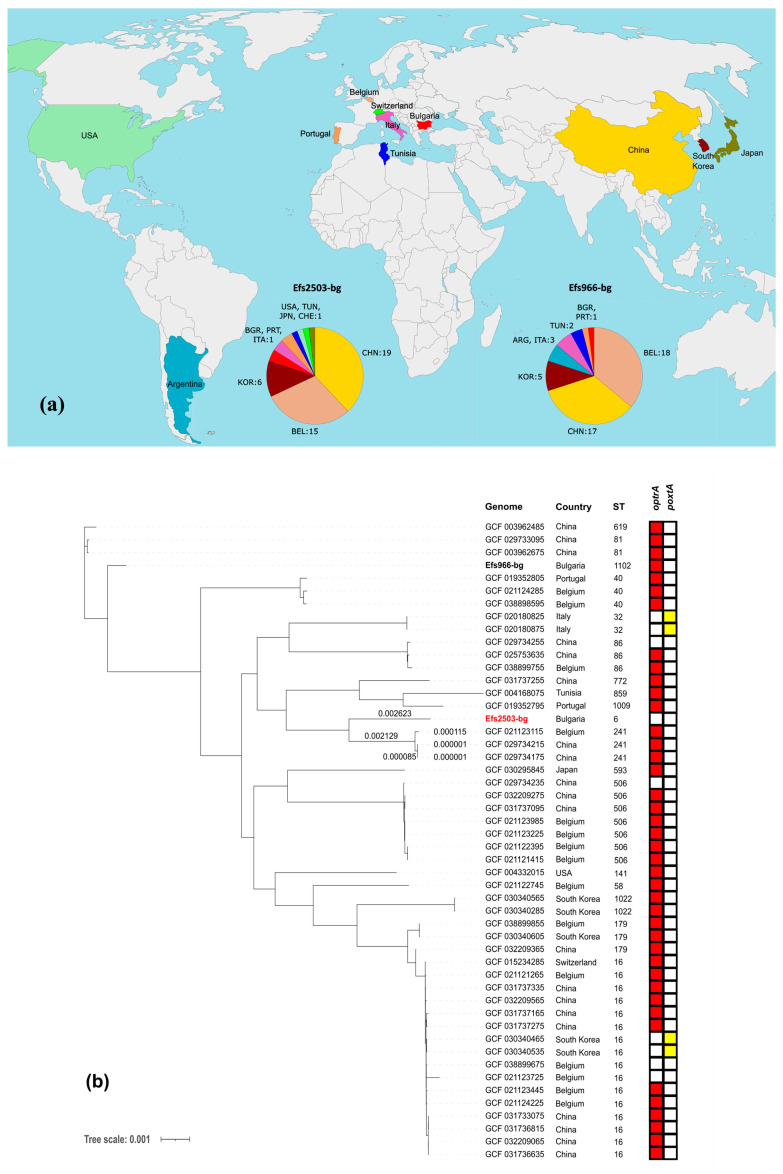

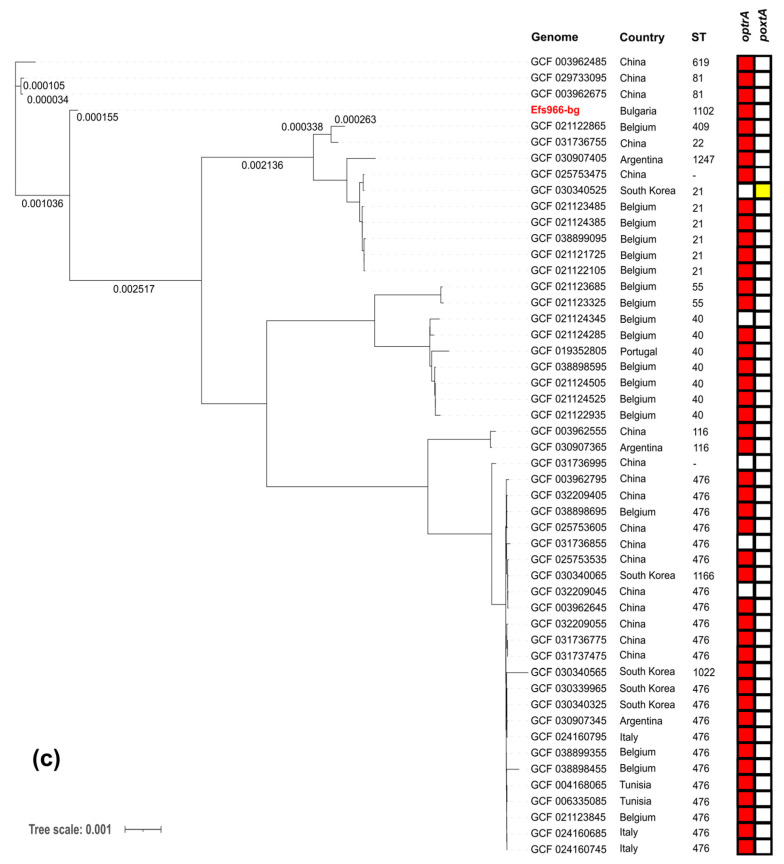
Phylogenomic analysis of linezolid-resistant *E. faecalis* strains with available genomes in the NCBI Nucleotide Database. (**a**) Geographical origins of the isolates included. Countries displayed in the diagram are labeled with their respective three-letter ISO codes, as specified by the ISO 3166 international standard [60]; (**b**,**c**) phylogenomic trees constructed by calling SNPs from the core gene alignment of linezolid-resistant *E. faecalis* isolates from Africa, Asia, Europe, South America, and North America (each comprising 50 isolates). A presence/absence matrix of linezolid resistance-related genes is also given (only for genes found at least in one isolate).

Next, a SNP distance matrix was constructed for all isolates based on sequence variants in the core gene alignment, as depicted above (Supplementary Table S2). The Efs2503-bg isolate exhibited a minimum of 6929 SNPs and a maximum of 19,092 SNPs when compared to the other isolates. For Efs966-bg, the corresponding values were 2198 SNPs and 18,668 SNPs, respectively. After sorting the matrix, the 50 strains most closely related to each Bulgarian *E. faecalis* isolate studied were identified (Figure 5a) and used to construct two phylogenetic trees based on SNPs found in the core gene alignment. The result is shown in Figure 5b,c.

Phylogenomic analysis revealed that Efs2503-bg and Efs966-bg are genetically distinct, with a difference of 12,051 SNPs. The closest to them are the Chinese strains GCF_029734175.1 and GCF_003962675.1, respectively.

## 4. Discussion

Linezolid (LNZ), the first oxazolidinone approved for clinical use in human medicine, has demonstrated undisputable clinical benefits in the treatment of serious infections caused by problematic Gram-positive cocci, including MDR and vancomycin-resistant *Enterococcus* spp. [61]. The global prevalence of LNZ resistance among these pathogens is considered to be low, as demonstrated by the ZyvoxVR Annual Appraisal of Potency and Spectrum (ZAAPS) program (2016). All tested *E. faecalis* isolates collected from Canada, Europe, Latin America, and the Asia-Pacific region demonstrated a MIC value of ≤4 mg/L, and a total of 99.8% of all Gram-positive pathogens were found to be susceptible to LNZ [62]. Nonetheless, a recent systematic review and meta-analysis of 43 studies from around the world, involving over 70,000 *E. faecalis* strains, reported an overall LNZ resistance rate of 2.2% (95% CI: 1.5–2.8). Furthermore, the prevalence of LNZ-resistant *E. faecalis* (LREfs) isolates was found to be 2.8% (95% CI: 1.9–3.7%) in Asia, 2.1% (95% CI: 0.6–3.6%) in Europe, and 0.7% (95% CI: 0.0–2.0%) in the Americas, showing higher resistance rates in certain geographic regions [63]. This concerning trend is further supported by data from the German Antimicrobial Resistance Surveillance program, which reveal a doubling in the rate of LRE among invasive isolates over a two-year period, increasing from 0.6% in 2019 to 1.2% in 2021 [64]. In the most recent meta-analysis, which summarizes the results of 84 studies from 2004 to 2021, the pooled prevalence of LRE, including both human and animal isolates, was estimated at 3.3% (95% CI; 2.3–4.6%) [65]. Among the human *Enterococcus* spp. isolates, the overall rate of LRE was 1.9%, which is similar to data from earlier studies. Moreover, the authors reported *E. faecalis* as the predominant LNZ-resistant species.

LREfs isolates of clinical origin in Europe have been reported in Spain [29], France [66], Poland [67], Belgium [68], the Czech Republic [69], Greece [70], and Ireland [36], among other locations. These studies demonstrate a higher prevalence of linezolid-resistant *E. faecium* (80–90%) compared to LREfs isolates (10–20%) with a single exception: the study of LRE isolated between 2013 and 2021 and received at the Belgian National Reference Centre (NRC) for Enterococci yielded 63 (81%) *E. faecalis* and 15 (19%) *E. faecium* strains, which were resistant to LNZ.

In this study, we report the first detection of clinical LREfs isolates in Bulgaria. It is important to note that the isolation of Efs2503-bg was preceded by a 3-week administration of LNZ, which is consistent with the findings of other authors. As presented in a recent systematic review, patients with LRE had been predominantly exposed to LNZ prior to isolation, with a mean treatment duration of 26.6 ± 46.1 days for *E. faecalis* and 25.7 ± 23.1 days for *E. faecium* [71]. The authors also reported that LRE could also develop in patients without prior LNZ exposure, as was the case with the Efs966-bg isolate obtained from an immunocompromised outpatient.

The application of WGS to AMR monitoring provides a deep understanding of the genetic mechanisms involved in the LNZ resistance among *Enterococcus* spp. These include mutations affecting a different number of 23S rDNA loci, as well as the presence of acquired LNZ resistance genes. Notably, the acquisition of the G2576T variant in the 23S rDNA, observed in our Efs2503-bg isolate, was not the predominant mechanism of LNZ resistance in *E. faecalis*, although it is widespread in other Garm-positive species. It was reported in 12 LREfs isolates from Spain, where 11 exhibited a 3:1 ratio of mutated to non-mutated loci, while the remaining isolate had a 1:1 ratio [72]. Like Efs2503-bg, 83.3% of them were isolated from patients who had received LNZ as part of their therapy with a median time of 7 days (3–13 days). This observation suggests that prolonged treatments with oxazolidinones could lead to spontaneous mutations in 23S rDNA. Despite the varying number of mutated loci in the Spanish isolates, they all exhibited LNZ MIC values of 128 mg/L, contrasting with an earlier report that suggested a direct correlation between the number of alleles harboring the G2576T mutation and the level of LNZ resistance [73]. Similar findings were reported in the Polish study, where LREfs isolates with two and three mutations affecting the same 23S rDNA loci exhibited identical resistance levels (32 mg/L) [67].

Analysis of the 332 LREfs genomes identified in our study revealed that the G2576T mutation was detected in only two complete and three draft genomes, confirming its low prevalence in this species. It is worth noting that the majority of these sequences were draft genomes assembled from short sequencing reads, which means that AMR-related mutations affecting only one or two copies of multicopy genes, such as the 23S rDNA, may be missed when assemblers collapse these sequences into a single copy [74,75].

In contrast to the G2576T mutation, found in Efs2503-bg, the C2163T variant was detected in only one of the four 23S rDNA loci of Efs966-bg. It was recently described as related to LNZ resistance in *Staphylococcus capitis* isolates obtained from a tertiary hospital in China [76] and has not yet been reported in enterococci. The contribution of the C2163T mutation to the LNZ resistance phenotype of Efs966-bg is challenging to assess, as this isolate also carries an *optrA* resistance determinant. The lower resistance level (8 mg/L), combined with the presence of an additional resistance mechanism, suggests a minimal, if any, contribution. Nevertheless, its discovery illustrates the fact that enterococci can accumulate mutations in their 23S rDNA even without prior exposure to LNZ.

The *optrA* gene has been detected in LRE from Asia, Europe, North America, and South America [65,77]. According to a recent systematic review and meta-analysis on the global occurrence of LRE, the *optrA* gene is the most frequently reported LNZ resistance mechanism in *E. faecalis* [65]. This finding was corroborated by the resistome analysis of *E. faecalis* strains included in our phylogenomic study, where 88.25% were found to be *optrA*-positive.

The *optrA* genetic environment in Efs966-bg encompasses the *fexA* gene, which facilitates the export of phenicols, an *ermA* determinant conferring resistance to macrolide-lincosamide-streptogramin B antibiotics, and *impB* (type VI secretion ImpB protein). This combination is commonly observed in the vicinity of *optrA* on medium-sized plasmids (30–60 kb), as previously reported [33]. Our analysis revealed that Efs966-bg harbors a plasmid closely resembling pAR0780 (CP063981.1; length: 65,096 bp). Notably, this plasmid belongs to the *rep*_9_ family and carries all three key sex-pheromone-response genes (*prgA*, *prgB*, and *prgC*). These findings indicate its potential for widespread dissemination via sex pheromone-mediated plasmid transfer, as proven before [78].

Two AME genes, responsible for HLAR (*aac(6′)-Ie-aph(2”)-Ia* and *aad6/ant(6)-Ia*), were detected in the MDR Efs2503-bg isolate. Niu et al. identified these determinants as the most prevalent AME genes among nosocomial isolates exhibiting high-level resistance to gentamicin and streptomycin in China [79]. Similar findings have also been reported in Iran [80] and Malaysia [81]. Moreover, according to the screening for AME genes among intestinal VRE obtained from critically ill patients at the hospital where Efs2503-bg was recovered, 88.9% of the isolates tested were positive for *aac(6′)-Ie-aph(2”)-Ia* [82].

Consistent with the clinical backgrounds of the patients from whom they were isolated, the investigated LREfs isolates exhibited some differences in their virulence gene profiles. The nosocomial Efs2503-bg isolate contains the complete repertoire of *cps* operon genes associated with capsule formation. As proven before, the presence of *cpsC*, *cpsD*, *cpsE*, *cpsG*, and *cpsI* is essential for the production of the high-molecular-weight capsular polysaccharide that contributes to pathogenesis through the evasion of the host innate immune system [83]. The existence of a capsule enhances the pathogenic potential of Efs2503-bg, aligning with its classification as part of the hospital-adapted ST6 lineage [84]. Notably, a representative strain of ST6 is the clinical vancomycin-resistant *E. faecalis* V583, the first *Enterococcus* to have its genome fully sequenced [85]. Moreover, ST6 belongs to the high-risk clonal complex (CC) 2, a group predominantly comprising hospital-associated isolates and characterized by the highest proportion of MDR strains [84]. Interestingly, several more LREfs ST6 isolates have been described in the literature, including 6 from Poland [67] and 1 from the Czech Republic [69]. All of these isolates harbor G2576T mutations in their 23S rDNA loci, without any additional transferable determinants associated with LNZ resistance, suggesting that the capsule may indeed act as a barrier to the transfer of plasmid-borne *optrA* genes.

The Efs966-bg isolate lacks all the essential *cps* genes needed to produce capsular polysaccharides. In contrast, its virulome harbors two copies of the *prgB* gene, one of which is located on the *optrA*-carrying plasmid. Manual analysis revealed that the plasmid also contains *prgA* and *prgC.* These three genes encode products that contribute to plasmid transfer, biofilm formation and virulence [86]. Their presence, coupled with the absence of a capsule, creates favorable conditions for sex pheromone-mediated plasmid transfer, as previously mentioned. This provides a plausible explanation for how the *optrA* gene was transferred to an ST1102 isolate, given that only two *E. faecalis* isolates from cold-water fish in Altay (China) have been reported so far with this ST, none of which was LNZ-resistant [87]. This makes Efs966-bg the first clinical isolate of this ST identified globally, as well as the first one to harbor an *optrA* gene. Its detection highlights the ability of *E. faecalis* isolates from diverse hosts to adapt to new environments and disseminate LNZ resistance-related genetic determinants through plasmid transfer in production practice and the food chain.

The significance of *optrA* transfer between different *Enterococcus* isolates became even more apparent from the resistome analysis of the *E. faecalis* strains subjected to phylogenomic analysis. This determinant was present in 88.3% of the strains. Importantly, a total of 70 different STs were detected, with the most dominant ones comprising less than 20% of the isolates. This observation suggests that the spread of LNZ resistance is more dependent on the transfer of *optrA*-carrying plasmids between isolates than on the expansion of specific STs with a large number of resistant isolates. Specific STs are relevant only if they group isolates that are more likely to possess barriers to plasmid transfer, such as capsule formation or CRISPR-Cas systems, or, conversely, lack such features. This hypothesis is further supported by the phylogenomic analysis, which revealed that most isolates were significantly different from each other, often showing more than 1000 SNP differences in their core genome alignments. That stands true also for Efs2503-bg and Efs966-bg, which, although originating from geographical regions in Bulgaria, differ by 12,051 SNPs.

In conclusion, to the best of our knowledge, this is the first detection of LRE associated with both hospital-acquired and community-acquired infections in Bulgaria. Furthermore, this is the first report worldwide of the isolation of human *optrA*-positive LREfs belonging to the rare ST1102. The presence of *optrA* was found to be the major cause of LNZ resistance in *E. faecalis* strains subjected to phylogenomic analysis. The obtained results emphasize the urgent need for surveillance measures of *optrA*-carrying *E. faecalis* isolates worldwide. Additionally, more efforts should be made to investigate the plasmidome of this species, as well as the involved mechanisms of plasmid transfer.

Although the global incidence of LRE is still low, their emergence is alarming and poses a growing clinical threat to public health. Continuous monitoring of antimicrobial resistance and screening for LRE intestinal colonization in high-risk patients, as well as their epidemiological typing, should be the mainstay of infection control stewardship practices in hospital settings.

## Figures and Tables

**Table 1:** Antimicrobial susceptibility testing of the studied linezolid-resistant *E. faecalis* isolates from Bulgaria.

Antimicrobial Agents	MIC [mg/L] and Interpretation	
	Efs2503-bg	Efs966-bg
Linezolid ^a^	>256 R	8 R
Ampicillin	0.50 S	0.75 S
Imipenem	1 I	0.75 I
Gentamicin (HLGR)	>256 (Positive)	8 (Negative)
Tigecycline	0.047 S	0.064 S
Eravacycline	0.08 S	0.016 S
Levofloxacin	>32 R	0.75 S
Vancomycin	0.75 S	0.50 S
Teicoplanin	0.25 S	0.50 S

MIC: minimum inhibitory concentration; R: resistant; I: intermediate (susceptible at increased exposure); S: susceptible; HLGR: high-level gentamicin resistance. ^a^ The MIC values determined by broth microdilution testing were >16 mg/L (Efs2503-bg) and 8 mg/L (Efs966-bg).

**Table 2:** Whole genome-based characterization of the studied linezolid-resistant *E. faecalis* isolates.

Isolate	Genome Size (Mb)	GC%	N50 (bp)	Number of Contigs (>1000 bp)	ST	Alleles						
						*gdh*	*gyd*	*pstS*	*gki*	*aroE*	*xpt*	*yqiL*
Efs2503-bg	3.18	37.23	331,629	67	6	1	2	16	28	26	2	1
Efs966-bg	2.97	37.20	1,457,140	8	1102	12	7	3	7	6	1	5

## Data Availability

The assembled genomes of the *E. faecalis* Efs2503-bg and Efs966-bg isolates were deposited in GenBank under BioProject accession number PRJNA1186976 (assemblies JBJHRT000000000 and JBJHRS000000000, respectively). The raw sequencing reads were submitted to the SRA database under BioProject PRJNA1186976.

## Supplementary Materials

The following supporting information can be downloaded at: https://www.mdpi.com/article/10.3390/microorganisms13010195/s1: Table S1: Genomes of linezolid-resistant *E. faecalis* isolates (*n* = 332) used for phylogenomic analysis: Sequence types and antimicrobial resistance determinants; Table S2: A SNP distance matrix based on sequence variants in the core gene alignment of all linezolid-resistant *E. faecalis* genomes used for phylogenomic analysis.

## References

[1] Dubin K., Pamer E.G. (2017). Enterococci and Their Interactions with the Intestinal Microbiome. Microbiol. Spectr..

[2] Sengupta M., Sarkar S., SenGupta M., Ghosh S., Sarkar R., Banerjee P. (2021). Biofilm Producing *Enterococcus* Isolates from Vaginal Microbiota. Antibiotics.

[3] Komiyama E.Y., Lepesqueur L.S.S., Yassuda C.G., Samaranayake L.P., Parahitiyawa N.B., Balducci I., Koga-Ito C.Y. (2016). *Enterococcus* Species in the Oral Cavity: Prevalence, Virulence Factors and Antimicrobial Susceptibility. PLoS ONE.

[4] Krawczyk B., Wityk P., Gałęcka M., Michalik M. (2021). The Many Faces of *Enterococcus* spp.—Commensal, Probiotic and Opportunistic Pathogen. Microorganisms.

[5] García-Solache M., Rice L.B. (2019). The *Enterococcus*: A Model of Adaptability to Its Environment. Clin. Microbiol. Rev..

[6] Brinkwirth S., Ayobami O., Eckmanns T., Markwart R. (2021). Hospital-Acquired Infections Caused by Enterococci: A Systematic Review and Meta-Analysis, WHO European Region, 1 January 2010 to 4 February 2020. Euro Surveill..

[7] Fiore E., Van Tyne D., Gilmore M.S. (2019). Pathogenicity of Enterococci. Microbiol. Spectr..

[8] Strateva T., Atanasova D., Savov E., Petrova G., Mitov I. (2016). Incidence of Virulence Determinants in Clinical *Enterococcus faecalis* and *Enterococcus faecium* Isolates Collected in Bulgaria. Braz. J. Infect. Dis..

[9] Kramer T.S., Remschmidt C., Werner S., Behnke M., Schwab F., Werner G., Gastmeier P., Leistner R. (2018). The Importance of Adjusting for *Enterococcus* Species When Assessing the Burden of Vancomycin Resistance: A Cohort Study Including over 1000 Cases of Enterococcal Bloodstream Infections. Antimicrob. Resist. Infect. Control.

[10] Puchter L., Chaberny I.F., Schwab F., Vonberg R.-P., Bange F.-C., Ebadi E. (2018). Economic Burden of Nosocomial Infections Caused by Vancomycin-Resistant Enterococci. Antimicrob. Resist. Infect. Control.

[11] Eichel V.M., Last K., Brühwasser C., Von Baum H., Dettenkofer M., Götting T., Grundmann H., Güldenhöven H., Liese J., Martin M. (2023). Epidemiology and Outcomes of Vancomycin-Resistant *Enterococcus* Infections: A Systematic Review and Meta-Analysis. J. Hosp. Infect..

[12] Tacconelli E., Carrara E., Savoldi A., Harbarth S., Mendelson M., Monnet D.L., Pulcini C., Kahlmeter G., Kluytmans J., Carmeli Y. (2018). Discovery, Research, and Development of New Antibiotics: The WHO Priority List of Antibiotic-Resistant Bacteria and Tuberculosis. Lancet Infect. Dis..

[13] Zahedi Bialvaei A., Rahbar M., Yousefi M., Asgharzadeh M., Samadi Kafil H. (2017). Linezolid: A Promising Option in the Treatment of Gram-Positives. J. Antimicrob. Chemother..

[14] Gould K. (2012). Clinical Update on Linezolid in the Treatment of Gram-Positive Bacterial Infections. IDR.

[15] Santimaleeworagun W., Changpradub D., Hemapanpairoa J., Thunyaharn S. (2021). Optimization of Linezolid Dosing Regimens for Treatment of Vancomycin-Resistant Enterococci Infection. Infect. Chemother..

[16] Bender J.K., Fleige C., Lange D., Klare I., Werner G. (2018). Rapid Emergence of Highly Variable and Transferable Oxazolidinone and Phenicol Resistance Gene *optrA* in German *Enterococcus* spp. Clinical Isolates. Int. J. Antimicrob. Agents.

[17] Bagga B., Buckingham S., Arnold S., Nesbitt A., Guimera D., Lee K. (2018). Increasing Linezolid-Resistant *Enterococcus* in a Children’s Hospital. Pediatr. Infect. Dis. J..

[18] Egan S.A., Corcoran S., McDermott H., Fitzpatrick M., Hoyne A., McCormack O., Cullen A., Brennan G.I., O’Connell B., Coleman D.C. (2020). Hospital Outbreak of Linezolid-Resistant and Vancomycin-Resistant ST80 *Enterococcus faecium* Harbouring an *optrA*-Encoding Conjugative Plasmid Investigated by Whole-Genome Sequencing. J. Hosp. Infect..

[19] Olearo F., Both A., Belmar Campos C., Hilgarth H., Klupp E.-M., Hansen J.L., Maurer F.P., Christner M., Aepfelbacher M., Rohde H. (2021). Emergence of Linezolid-Resistance in Vancomycin-Resistant *Enterococcus faecium* ST117 Associated with Increased Linezolid-Consumption. Int. J. Med. Microbiol..

[20] Hasman H., Clausen P.T.L.C., Kaya H., Hansen F., Knudsen J.D., Wang M., Holzknecht B.J., Samulioniené J., Røder B.L., Frimodt-Møller N. (2019). LRE-Finder, a Web Tool for Detection of the 23S rRNA Mutations and the *optrA*, *cfr*, *cfr(B)* and *poxtA* Genes Encoding Linezolid Resistance in Enterococci from Whole-Genome Sequences. J. Antimicrob. Chemother..

[21] Bender J.K., Cattoir V., Hegstad K., Sadowy E., Coque T.M., Westh H., Hammerum A.M., Schaffer K., Burns K., Murchan S. (2018). Update on Prevalence and Mechanisms of Resistance to Linezolid, Tigecycline and Daptomycin in Enterococci in Europe: Towards a Common Nomenclature. Drug Resist. Updat..

[22] Lee S.-M., Huh H.J., Song D.J., Shim H.J., Park K.S., Kang C.-I., Ki C.-S., Lee N.Y. (2017). Resistance Mechanisms of Linezolid-Nonsusceptible Enterococci in Korea: Low Rate of 23S rRNA Mutations in *Enterococcus faecium*. J. Med. Microbiol..

[23] Schwarz S., Werckenthin C., Kehrenberg C. (2000). Identification of a Plasmid-Borne Chloramphenicol-Florfenicol Resistance Gene in *Staphylococcus sciuri*. Antimicrob. Agents Chemother..

[24] Shen J., Wang Y., Schwarz S. (2013). Presence and Dissemination of the Multiresistance Gene *cfr* in Gram-Positive and Gram-Negative Bacteria. J. Antimicrob. Chemother..

[25] Long K.S., Poehlsgaard J., Kehrenberg C., Schwarz S., Vester B. (2006). The Cfr rRNA Methyltransferase Confers Resistance to Phenicols, Lincosamides, Oxazolidinones, Pleuromutilins, and Streptogramin A Antibiotics. Antimicrob. Agents Chemother..

[26] Bender J.K., Fleige C., Klare I., Fiedler S., Mischnik A., Mutters N.T., Dingle K.E., Werner G. (2016). Detection of a *cfr(B)* Variant in German *Enterococcus faecium* Clinical Isolates and the Impact on Linezolid Resistance in *Enterococcus* spp.. PLoS ONE.

[27] Kuroda M., Sekizuka T., Matsui H., Suzuki K., Seki H., Saito M., Hanaki H. (2018). Complete Genome Sequence and Characterization of Linezolid-Resistant *Enterococcus faecalis* Clinical Isolate KUB3006 Carrying a *cfr(B)*-Transposon on Its Chromosome and *optrA*-Plasmid. Front. Microbiol..

[28] Guerin F., Sassi M., Dejoies L., Zouari A., Schutz S., Potrel S., Auzou M., Collet A., Lecointe D., Auger G. (2020). Molecular and Functional Analysis of the Novel *cfr(D)* Linezolid Resistance Gene Identified in *Enterococcus faecium*. J. Antimicrob. Chemother..

[29] Ruiz-Ripa L., Feßler A.T., Hanke D., Eichhorn I., Azcona-Gutiérrez J.M., Pérez-Moreno M.O., Seral C., Aspiroz C., Alonso C.A., Torres L. (2020). Mechanisms of Linezolid Resistance Among Enterococci of Clinical Origin in Spain—Detection of *optrA-* and *cfr(D)*-Carrying *E. faecalis*. Microorganisms.

[30] Sharkey L.K.R., Edwards T.A., O’Neill A.J. (2016). ABC-F Proteins Mediate Antibiotic Resistance through Ribosomal Protection. mBio.

[31] Fu Y., Deng Z., Shen Y., Wei W., Xiang Q., Liu Z., Hanf K., Huang S., Lv Z., Cao T. (2024). High Prevalence and Plasmidome Diversity of *optrA*-Positive Enterococci in a Shenzhen Community, China. Front. Microbiol..

[32] Wang Y., Lv Y., Cai J., Schwarz S., Cui L., Hu Z., Zhang R., Li J., Zhao Q., He T. (2015). A Novel Gene, *optrA*, That Confers Transferable Resistance to Oxazolidinones and Phenicols and Its Presence in *Enterococcus faecalis* and *Enterococcus faecium* of Human and Animal Origin. J. Antimicrob. Chemother..

[33] Freitas A.R., Tedim A.P., Novais C., Lanza V.F., Peixe L. (2020). Comparative Genomics of Global *optrA*-Carrying *Enterococcus faecalis* Uncovers a Common Chromosomal Hotspot for *optrA* Acquisition within a Diversity of Core and Accessory Genomes. Microb. Genom..

[34] Antonelli A., D’Andrea M.M., Brenciani A., Galeotti C.L., Morroni G., Pollini S., Varaldo P.E., Rossolini G.M. (2018). Characterization of *poxtA*, a Novel Phenicol–Oxazolidinone–Tetracycline Resistance Gene from an MRSA of Clinical Origin. J. Antimicrob. Chemother..

[35] Papagiannitsis C.C., Tsilipounidaki K., Malli E., Petinaki E. (2019). Detection in Greece of a Clinical *Enterococcus faecium* Isolate Carrying the Novel Oxazolidinone Resistance Gene *poxtA*. J. Antimicrob. Chemother..

[36] Egan S.A., Shore A.C., O’Connell B., Brennan G.I., Coleman D.C. (2020). Linezolid Resistance in *Enterococcus faecium* and *Enterococcus faecalis* from Hospitalized Patients in Ireland: High Prevalence of the MDR Genes *optrA* and *poxtA* in Isolates with Diverse Genetic Backgrounds. J. Antimicrob. Chemother..

[37] Freitas A.R., Tedim A.P., Duarte B., Elghaieb H., Abbassi M.S., Hassen A., Read A., Alves V., Novais C., Peixe L. (2020). Linezolid-Resistant (Tn *6246* :: *fexB*–*poxtA* ) *Enterococcus faecium* Strains Colonizing Humans and Bovines on Different Continents: Similarity without Epidemiological Link. J. Antimicrob. Chemother..

[38] Rodriguez-R L.M., Gunturu S., Harvey W.T., Rosselló-Mora R., Tiedje J.M., Cole J.R., Konstantinidis K.T. (2018). The Microbial Genomes Atlas (MiGA) Webserver: Taxonomic and Gene Diversity Analysis of Archaea and Bacteria at the Whole Genome Level. Nucleic Acids Res..

[39] The European Committee on Antimicrobial Susceptibility Testing (EUCAST) (2024). Breakpoint Tables for Interpretation of MICs and Zone Diameters, Version 14.0. https://eucast.org.

[40] The European Committee on Antimicrobial Susceptibility Testing EUCAST Reading Guide for Broth Microdilution. https://www.eucast.org/fileadmin/src/media/PDFs/EUCAST_files/Disk_test_documents/2022_manuals/Reading_guide_BMD_v_4.0_2022.pdf.

[41] Magiorakos A.-P., Srinivasan A., Carey R.B., Carmeli Y., Falagas M.E., Giske C.G., Harbarth S., Hindler J.F., Kahlmeter G., Olsson-Liljequist B. (2012). Multidrug-Resistant, Extensively Drug-Resistant and Pandrug-Resistant Bacteria: An International Expert Proposal for Interim Standard Definitions for Acquired Resistance. Clin. Microbiol. Infect..

[42] Bolger A.M., Lohse M., Usadel B. (2014). Trimmomatic: A Flexible Trimmer for Illumina Sequence Data. Bioinformatics.

[43] Bankevich A., Nurk S., Antipov D., Gurevich A.A., Dvorkin M., Kulikov A.S., Lesin V.M., Nikolenko S.I., Pham S., Prjibelski A.D. (2012). SPAdes: A New Genome Assembly Algorithm and Its Applications to Single-Cell Sequencing. J. Comput. Biol..

[44] Gurevich A., Saveliev V., Vyahhi N., Tesler G. (2013). QUAST: Quality Assessment Tool for Genome Assemblies. Bioinformatics.

[45] Afgan E., Nekrutenko A., Grüning B.A., Blankenberg D., Goecks J., Schatz M.C., Ostrovsky A.E., Mahmoud A., Lonie A.J., The Galaxy Community (2022). The Galaxy Platform for Accessible, Reproducible and Collaborative Biomedical Analyses: 2022 Update. Nucleic Acids Res..

[46] Alcock B.P., Huynh W., Chalil R., Smith K.W., Raphenya A.R., Wlodarski M.A., Edalatmand A., Petkau A., Syed S.A., Tsang K.K. (2023). CARD 2023: Expanded Curation, Support for Machine Learning, and Resistome Prediction at the Comprehensive Antibiotic Resistance Database. Nucleic Acids Res..

[47] Langmead B., Salzberg S.L. (2012). Fast Gapped-Read Alignment with Bowtie 2. Nat. Methods.

[48] Robinson J.T., Thorvaldsdottir H., Turner D., Mesirov J.P. (2023). Igv.Js: An Embeddable JavaScript Implementation of the Integrative Genomics Viewer (IGV). Bioinformatics.

[49] Alikhan N.-F., Petty N.K., Ben Zakour N.L., Beatson S.A. (2011). BLAST Ring Image Generator (BRIG): Simple Prokaryote Genome Comparisons. BMC Genom..

[50] Liu B., Zheng D., Zhou S., Chen L., Yang J. (2022). VFDB 2022: A General Classification Scheme for Bacterial Virulence Factors. Nucleic Acids Res..

[51] Ruiz-Garbajosa P., Bonten M.J.M., Robinson D.A., Top J., Nallapareddy S.R., Torres C., Coque T.M., Cantón R., Baquero F., Murray B.E. (2006). Multilocus Sequence Typing Scheme for *Enterococcus faecalis* Reveals Hospital-Adapted Genetic Complexes in a Background of High Rates of Recombination. J. Clin. Microbiol..

[52] Parks D.H., Imelfort M., Skennerton C.T., Hugenholtz P., Tyson G.W. (2015). CheckM: Assessing the Quality of Microbial Genomes Recovered from Isolates, Single Cells, and Metagenomes. Genome Res..

[53] Seemann T. (2014). Prokka: Rapid Prokaryotic Genome Annotation. Bioinformatics.

[54] Page A.J., Cummins C.A., Hunt M., Wong V.K., Reuter S., Holden M.T.G., Fookes M., Falush D., Keane J.A., Parkhill J. (2015). Roary: Rapid Large-Scale Prokaryote Pan Genome Analysis. Bioinformatics.

[55] Stamatakis A. (2014). RAxML Version 8: A Tool for Phylogenetic Analysis and Post-Analysis of Large Phylogenies. Bioinformatics.

[56] Letunic I., Bork P. (2024). Interactive Tree of Life (iTOL) v6: Recent Updates to the Phylogenetic Tree Display and Annotation Tool. Nucleic Acids Res..

[57] Miller W.R., Munita J.M., Arias C.A. (2014). Mechanisms of Antibiotic Resistance in Enterococci. Expert. Rev. Anti. Infect. Ther..

[58] Sharifzadeh Peyvasti V., Mohabati Mobarez A., Shahcheraghi F., Khoramabadi N., Razaz Rahmati N., Hosseini Doust R. (2020). High-Level Aminoglycoside Resistance and Distribution of Aminoglycoside Resistance Genes among *Enterococcus* spp. Clinical Isolates in Tehran, Iran. J. Glob. Antimicrob. Resist..

[59] Ross K., Varani A.M., Snesrud E., Huang H., Alvarenga D.O., Zhang J., Wu C., McGann P., Chandler M. (2021). TnCentral: A Prokaryotic Transposable Element Database and Web Portal for Transposon Analysis. mBio.

[60] (1974). Codes for the Representation of Names of Countries and Their Subdivisions.

[61] Mendes R.E., Deshpande L.M., Jones R.N. (2014). Linezolid Update: Stable in Vitro Activity Following More than a Decade of Clinical Use and Summary of Associated Resistance Mechanisms. Drug Resist. Updat..

[62] Mendes R.E., Deshpande L., Streit J.M., Sader H.S., Castanheira M., Hogan P.A., Flamm R.K. (2018). ZAAPS Programme Results for 2016: An Activity and Spectrum Analysis of Linezolid Using Clinical Isolates from Medical Centres in 42 Countries. J. Antimicrob. Chemother..

[63] Dadashi M., Sharifian P., Bostanshirin N., Hajikhani B., Bostanghadiri N., Khosravi-Dehaghi N., Van Belkum A., Darban-Sarokhalil D. (2021). The Global Prevalence of Daptomycin, Tigecycline, and Linezolid-Resistant *Enterococcus faecalis* and *Enterococcus faecium* Strains From Human Clinical Samples: A Systematic Review and Meta-Analysis. Front. Med..

[64] Bender J.K., Fleige C., Funk F., Moretó-Castellsagué C., Fischer M.A., Werner G. (2024). Linezolid Resistance Genes and Mutations among Linezolid-Susceptible *Enterococcus* spp.—A Loose Cannon?. Antibiotics.

[65] Wada Y., Afolabi H.A., Al-Mhanna S.B., Bello K.E., Irekeola A.A., Wada M., Ahmed N., Harun A., Yean C.Y., Mohamad Nasir N.S. (2024). Global Occurrence of Linezolid-Resistant *Enterococcus* (LRE): The First Systematic Review and Meta-Analysis. The Microbe.

[66] Sassi M., Guérin F., Zouari A., Beyrouthy R., Auzou M., Fines-Guyon M., Potrel S., Dejoies L., Collet A., Boukthir S. (2019). Emergence of *optrA*-Mediated Linezolid Resistance in Enterococci from France, 2006–2016. J. Antimicrob. Chemother..

[67] Gawryszewska I., Żabicka D., Hryniewicz W., Sadowy E. (2017). Linezolid-Resistant Enterococci in Polish Hospitals: Species, Clonality and Determinants of Linezolid Resistance. Eur. J. Clin. Microbiol. Infect. Dis..

[68] Mortelé O., Van Kleef–van Koeveringe S., Vandamme S., Jansens H., Goossens H., Matheeussen V. (2024). Epidemiology and Genetic Diversity of Linezolid-Resistant *Enterococcus* Clinical Isolates in Belgium from 2013 to 2021. J. Glob. Antimicrob. Resist..

[69] Mališová L., Jakubů V., Pomorská K., Musílek M., Žemličková H. (2021). Spread of Linezolid-Resistant *Enterococcus* spp. in Human Clinical Isolates in the Czech Republic. Antibiotics.

[70] Tsilipounidaki K., Gerontopoulos A., Papagiannitsis C., Petinaki E. (2019). First Detection of an *optrA*-Positive, Linezolid-Resistant ST16 *Enterococcus faecalis* from Human in Greece. New Microbes New Infect..

[71] Bi R., Qin T., Fan W., Ma P., Gu B. (2018). The Emerging Problem of Linezolid-Resistant Enterococci. J. Glob. Antimicrob. Resist..

[72] Gómez-Gil R., Romero-Gómez M.P., García-Arias A., Ubeda M.G., Busselo M.S., Cisterna R., Gutiérrez-Altés A., Mingorance J. (2009). Nosocomial Outbreak of Linezolid-Resistant *Enterococcus faecalis* Infection in a Tertiary Care Hospital. Diagn. Microbiol. Infect. Dis..

[73] Ruggero K.A., Schroeder L.K., Schreckenberger P.C., Mankin A.S., Quinn J.P. (2003). Nosocomial Superinfections Due to Linezolid-Resistant *Enterococcus faecalis*: Evidence for a Gene Dosage Effect on Linezolid MICs. Diagn. Microbiol. Infect. Dis..

[74] Sinclair A., Arnold C., Woodford N. (2003). Rapid Detection and Estimation by Pyrosequencing of 23S rRNA Genes with a Single Nucleotide Polymorphism Conferring Linezolid Resistance in Enterococci. Antimicrob. Agents Chemother..

[75] Ellington M.J., Ekelund O., Aarestrup F.M., Canton R., Doumith M., Giske C., Grundman H., Hasman H., Holden M.T.G., Hopkins K.L. (2017). The Role of Whole Genome Sequencing in Antimicrobial Susceptibility Testing of Bacteria: Report from the EUCAST Subcommittee. Clin. Microbiol. Infect..

[76] Zhou W., Niu D., Gao S., Zhong Q., Liu C., Liao X., Cao X., Zhang Z., Zhang Y., Shen H. (2023). Prevalence, Biofilm Formation, and Mass Spectrometric Characterization of Linezolid-Resistant *Staphylococcus capitis* Isolated from a Tertiary Hospital in China. J. Glob. Antimicrob. Resist..

[77] Wang Z., Liu D., Zhang J., Liu L., Zhang Z., Liu C., Hu S., Wu L., He Z., Sun H. (2024). Genomic Epidemiology Reveals Multiple Mechanisms of Linezolid Resistance in Clinical Enterococci in China. Ann. Clin. Microbiol. Antimicrob..

[78] Zou J., Tang Z., Yan J., Liu H., Chen Y., Zhang D., Zhao J., Tang Y., Zhang J., Xia Y. (2020). Dissemination of Linezolid Resistance Through Sex Pheromone Plasmid Transfer in *Enterococcus faecalis*. Front. Microbiol..

[79] Niu H., Yu H., Hu T., Tian G., Zhang L., Guo X., Hu H., Wang Z. (2016). The Prevalence of Aminoglycoside-Modifying Enzyme and Virulence Genes among Enterococci with High-Level Aminoglycoside Resistance in Inner Mongolia, China. Braz. J. Microbiol..

[80] Amini F., Krimpour H.A., Ghaderi M., Vaziri S., Ferdowsi S., Azizi M., Amini S. (2018). Prevalence of Aminoglycoside Resistance Genes in *Enterococcus* Strains in Kermanshah, Iran. Iran. J. Med. Sci..

[81] Moussa A.A., Md Nordin A.F., Hamat R.A., Jasni A.S. (2019). High Level Aminoglycoside Resistance And Distribution Of The Resistance Genes In *Enterococcus faecalis* And *Enterococcus faecium* From Teaching Hospital in Malaysia. IDR.

[82] Hristova P.M., Nankov V.M., Stoikov I., Nikolaev Ivanov I., Vaskova Ouzounova-Raykova V., Hitkova H.Y. (2022). Prevalence of Genes Encoding Resistance to Aminoglycosides and Virulence Factors Among Intestinal Vancomycin-Resistant Enterococci. Jundishapur. J. Microbiol..

[83] Thurlow L.R., Thomas V.C., Hancock L.E. (2009). Capsular Polysaccharide Production in *Enterococcus faecalis* and Contribution of *cpsF* to Capsule Serospecificity. J. Bacteriol..

[84] Kuch A., Willems R.J.L., Werner G., Coque T.M., Hammerum A.M., Sundsfjord A., Klare I., Ruiz-Garbajosa P., Simonsen G.S., Van Luit-Asbroek M. (2012). Insight into Antimicrobial Susceptibility and Population Structure of Contemporary Human *Enterococcus faecalis* Isolates from Europe. J. Antimicrob. Chemother..

[85] Paulsen I.T., Banerjei L., Myers G.S.A., Nelson K.E., Seshadri R., Read T.D., Fouts D.E., Eisen J.A., Gill S.R., Heidelberg J.F. (2003). Role of Mobile DNA in the Evolution of Vancomycin-Resistant *Enterococcus faecalis*. Science.

[86] Bhatty M., Cruz M.R., Frank K.L., Laverde Gomez J.A., Andrade F., Garsin D.A., Dunny G.M., Kaplan H.B., Christie P.J. (2015). *Enterococcus faecalis* pCF 10-encoded Surface Proteins PrgA, PrgB (Aggregation Substance) and PrgC Contribute to Plasmid Transfer, Biofilm Formation and Virulence. Mol. Microbiol..

[87] Xueling Z., Lixia Y., Huimin Z., Fengwei T., Yongqing N.I. (2022). Clonal Relationships among *Enterococcus faecalis* from Humans and Animal-Origin Foods in Xinjiang Characterized by Multilocus Sequence Typing. Food Sci..

